# Cognitive and physiological effects of an acute physical activity intervention in elementary school children

**DOI:** 10.3389/fpsyg.2014.01473

**Published:** 2014-12-18

**Authors:** Katja Jäger, Mirko Schmidt, Achim Conzelmann, Claudia M. Roebers

**Affiliations:** ^1^Department of Psychology, University of BernBern, Switzerland; ^2^Institute of Sport Science, University of BernBern, Switzerland

**Keywords:** cognition, executive functions, acute exercise, intervention, salivary cortisol

## Abstract

The aim of the present study was to investigate the effects of an acute physical activity intervention that included cognitive engagement on executive functions and on cortisol level in young elementary school children. Half of the 104 participating children (6–8 years old) attended a 20-min sport sequence, which included cognitively engaging and playful forms of physical activity. The other half was assigned to a resting control condition. Individual differences in children's updating, inhibition, and shifting performance as well as salivary cortisol were assessed before (pre-test), immediately after (post-test), and 40 min after (follow-up) the intervention or control condition, respectively. Results revealed a significantly stronger improvement in inhibition in the experimental group compared to the control group, while it appeared that acute physical activity had no specific effect on updating and shifting. The intervention effect on inhibition leveled out 40 min after physical activity. Salivary cortisol increased significantly more in the experimental compared to the control group between post-test and follow-up and results support partly the assumed inverted U-shaped relationship between cortisol level and cognitive performance. In conclusion, results indicate that acute physical activity that includes cognitive engagement may have immediate positive effects on inhibition, but not necessarily on updating and shifting in elementary school children. This positive effect may partly be explained through cortisol elevation after acute physical activity.

## Introduction

Experimental research shows that exercise and acute physical activity not only affects physical development and health positively (Bailey, 2006), but may also enhance cognitive performance (Colcombe and Kramer, 2003; Hillman et al., 2008; Tomporowski et al., 2008b; Best, 2010; Chang et al., 2012). However, most studies in this area were conducted with adults and empirical investigations that include young school children are still rare. As a result, studies addressing the question whether exercise and acute physical activity may indeed yield cognitive improvements in young elementary school children are being called for (Diamond and Lee, 2011). Important, yet open questions in this context are, among others, the issue of which aspects of cognitive performance can be improved more easily than others and how long potential benefits may last. In addition, the physiological processes underlying the assumed effect of exercise and acute physical activity on cognitive performance are still unclear and studies including physiological measures are needed to address this issue.

The most promising aspect of cognitive functioning to be positively affected by exercise and acute physical activity in adults and children seems to be the domain of executive functions (EFs; Colcombe and Kramer, 2003; Tomporowski et al., 2011). EFs refer to the individual's ability to initiate, adapt, regulate, monitor, and control information processes and behavior (Miyake et al., 2000; Hughes and Graham, 2002; Diamond, 2013). In the literature, a division of EFs into three subdimensions is suggested: updating (keeping relevant information in working memory and processing this information by replacing old, no longer relevant information with newer, more relevant information), inhibition (ability to avoid dominant, automatic, or prepotent responses or resisting distractor interference as well as suppressing environmental interference), and shifting (moving back and forth between multiple tasks, operations, rules, or mental sets; Miyake et al., 2000; Friedman and Miyake, 2004; Diamond, 2013). The examination of different subdimension of EFs separately seems to be reasonable because it may increase our understanding of EFs and their development. In addition, the measurement of highly specific constructs may be more sensitive for detecting changes attributable to physical exercise or other factors than global measurements (Hillman et al., 2005; Tomporowski et al., 2008b).

Against the background of EFs being highly relevant for school achievement (Riggs et al., 2004; Blair and Diamond, 2008; Best et al., 2011; Roebers et al., 2011, 2014; Diamond, 2012) and the theoretical assumption of shared cognitive processes involved in both motor and cognitive control (Diamond, 2000), it is not surprising that the investigation of effects of exercise and acute physical activity on EFs recently has become an increasingly popular topic of research. Regarding effects of acute physical activity, several empirical studies with children or adolescents revealed positive intervention effects on inhibition (Hillman et al., 2009; Kubesch et al., 2009; Best, 2012; Drollette et al., 2012; Pontifex et al., 2012; Hogan et al., 2013). For updating and shifting, however, no (neither positive nor negative) effects have been documented so far (Tomporowski et al., 2008a; Kubesch et al., 2009; Budde et al., 2010a; Drollette et al., 2012). These results indicate a differential susceptibility of EFs, with inhibition being the subdimension benefitting more easily from acute physical activity compared to other subdimensions. However, the number of studies assessing the effects of acute physical activity on updating and shifting in young children is very limited and most studies did not assess all three EF subdimensions simultaneously. Thus, more research is needed to address this topic.

In addition, most studies to date have examined the effects of endurance-oriented interventions, i.e., employing treadmill running, ergometer cycling or other forms of aerobic endurance exercise that can be considered as not optimally suited for physical education lessons. To our knowledge, only two studies compared the effects of an acute physical activity intervention including some form of cognitive engagement (cognitively engaging exergaming, team games) to a non-active control condition (Pesce et al., 2009; Best, 2012). Subjects in these studies showed better cognitive performance (inhibition, free-recall memory) after the intervention than the control condition. More studies are needed to confirm these results and to extend knowledge about effects on other domains of cognitive functioning, such as updating and shifting. Furthermore, the effects of physical activities that include EF-specific cognitive engagement have been targeted only in a long-term intervention study, showing positive effects on attention in typically developing children (Pesce et al., 2013). To date, no study addressed the effects of an acute physical activity intervention including EF-specific cognitive engagement such as keeping in mind different rules, updating these rules, reacting appropriately to different rules, inhibiting prepotent movements, as well as shifting between different situations and rules. This form of acute physical activity might be particularly beneficial for EF performance as a result of the cognitive engagement in addition to the physical activation. Moreover, it might be somewhat more child-appropriate due to the game character and the implied fun aspect that is important when working with children (Diamond, 2012).

Another yet unanswered question concerns the temporal stability of potential positive effects of acute physical activity interventions. A recent meta-analysis (Chang et al., 2012) indicates that the strongest positive effects of acute physical activity on EFs in adults emerged after a delay of about 10–20 min. Investigations that include a follow-up measurement assessing longer-term benefits of acute physical activity in children are rare. In the study by Kubesch et al. (2009), the initial positive effects of 30 min aerobic exercise on inhibition in 13–14-year-old children were no longer evident after 45 min. Hillman et al. (2009) also concluded that beneficial effects of aerobic exercise on academic achievement seem to level out after about 1 h. Based on the few existing results it is not possible to draw well-grounded conclusions about the temporal stability of potential effects. This, however, is an important question for theory and practice.

There is still a lack of knowledge about physiological processes underlying the relationship between acute physical activity and cognitive performance. The following mechanisms are discussed to be responsible for effects of acute physical activity on cognitive functioning: an enhanced cerebral blood flow (Suzuki et al., 2004; Timinkul et al., 2008), the release of nerve growth factors and the release of brain-derived neurotrophic factors (Gold et al., 2003; Vaynman et al., 2004; Ferris et al., 2007; Winter et al., 2007), and/or the release of neuroendocrinological substances, such as epinephrine (Cahill and Alkire, 2003), dopamine (Winter et al., 2007), serotonin (Hollmann and Strüder, 2000), and cortisol (Blair et al., 2005).

The release of cortisol as an underlying mechanism is a promising approach since it seems to affect numerous cognitive domains (Erickson et al., 2003) and it is not only released due to psychosocial stress, but also through physical activity (Kirschbaum and Hellhammer, 1994; Papacosta and Nassis, 2011). Several studies with adults and adolescents showed an increased cortisol level after acute physical activity (Jacks et al., 2002; Di Luigi et al., 2006; Benitez-Sillero et al., 2009; Thomas et al., 2009; Budde et al., 2010a). Corresponding results in children, however, are somewhat less clear. One study (Budde et al., 2010b) with prepubescent children found no cortisol elevation after acute physical activity and another study (Benitez-Sillero et al., 2009) found an elevation only in physically fit children. A third study (Heijsman et al., 2012) reported no elevation when cortisol was assessed immediately after acute physical activity, but did so when it was assessed again 15 min later. This finding is in line with results showing that the peak of cortisol concentration occurs with a latency of 20–30 min following physical activity (Kirschbaum and Hellhammer, 1994; Daly et al., 2005). Taken together, acute physical activity seems to yield a delayed increase in cortisol concentration, although this finding still needs to be verified in children.

Cortisol affects different neuropeptide and neurotransmitter systems and therefore, it seems to influence various cognitive processes such as perception, selective attention, and memory (Erickson et al., 2003; Lupien et al., 2005). The relationship between cortisol and cognitive performance appears to follow an inverted U-shaped curve, with moderate elevations of cortisol concentration being associated with an improvement in cognitive performance, whereas highly elevated cortisol concentrations seem to be associated with impairments in cognitive functioning (Erickson et al., 2003; Blair et al., 2005).

To our knowledge, only one research group (Budde et al., 2010a) tested the assumption that cortisol might be an underlying mechanism contributing to the effect of acute physical activity on cognitive performance directly. They could, however, *not* confirm this assumption since they did not find a relationship between physical activity-related changes in working memory and cortisol concentration. Thus, by now, only weak hints for a relationship between cortisol elevation due to acute physical activity and cognitive performance exist.

The present exploratory study aimed to shed light on the following aspects regarding the effects of an acute physical activity intervention on young children's EFs and their cortisol level: (1) A particular paucity of results is evident regarding studies that examine cognitively engaging and playful forms of acute physical activity in young elementary school children. To address this paucity, we investigated the effects of a playful and cognitively engaging acute physical activity intervention on EFs in second-graders. (2) Because most existing studies did not assess all three EF subdimensions defined by Miyake et al. (2000) simultaneously, it is difficult to draw firm conclusions about which aspect of EFs is more easily affected by acute physical activity than others. Therefore, we included measures of updating, inhibition, and shifting in our study. (3) As it is not clear how long potential effects last after acute physical activity, we assessed the three EF subdimensions prior, immediately after, and as follow-up 40 min after the intervention and compared the children's performance with a resting control group. (4) Finally, we included measurements of cortisol level at all three measurement points in order to explore physiological processes occurring in relation to acute physical activity and cognitive performance.

## Methods

### Subjects

A total of 108 2nd-grade children from nine different classes in the region of Bern (Switzerland) participated in the study. Both the participants themselves and their parents gave consent to take part in the study. Ethical consent for the study was obtained by the local ethics committees. Of the original data set, data of the mixed block in the Flanker task from two children at pre-test and the entire data from four other children had to be excluded due to technical problems. The final sample consisted of 104 children (54.8% girls) aged 82–107 months (*M* = 94.91 months; *SD* = 5.05 months). Participants were randomly assigned to either an experimental group (*EG*; *n* = 51, 52.9% girls) or a control group (*CG*; *n* = 53, 56.6% girls).

### Procedure

**Figure 1:**
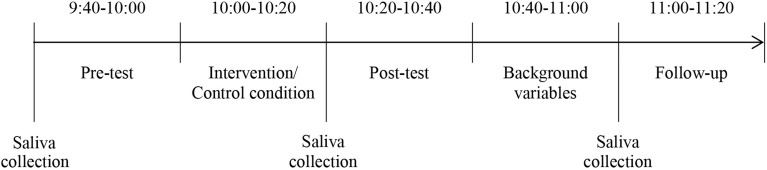
**Timeline of the study design**.

Each child completed the same cognitive testing three times in the course of one school morning: before (pre-test), immediately after (post-test) and 40 min after (follow-up) the intervention/control condition. Cognitive testing took place in a quiet room in individual settings. After the pre-test, children went to the gymnasium in groups of four to attend the intervention or control condition. Immediately after the intervention/control condition, participants completed the post-test, again in individual settings and with the same investigator as in the pre-test. Investigators were blind to the condition (experimental vs. control) children were allocated to. Subsequently, the children went back to the gymnasium where their enjoyment, their height, weight, socioeconomic status (SES) and activity level were recorded. Finally, the last trial of cognitive testing (follow-up) was completed. Each single block of testing and intervention/control condition took about 20 min. At the beginning of every cognitive testing, a saliva sample of each child was collected, whereof cortisol concentration was obtained. The timeline of the study design is depicted in Figure 1. Academic achievement (math, language) was estimated by the teacher at the same day as the intervention was conducted. Motor fitness was assessed independently during a physical education lesson 2–4 weeks after the intervention.

### Intervention/control condition

Both, the physical activity intervention and the control condition were implemented in the gymnasium of the school (the two conditions were conducted alternately, not at the same time). Children dressed for physical activity and were equipped with a wireless heart-rate monitor. They then attended the intervention or control condition. Afterwards, heart-rate monitors were removed and children went back to the room were the cognitive testing took place.

The acute physical activity intervention included EF-specific cognitive engagement. It started with a warm-up exercise of running during which children listened to a song. Whenever specific words were mentioned in the song, they had to carry out a certain movement (e.g., touch the floor, spin around, jump up) which was introduced before the song was played. This exercise was repeated three times with an increasing level of difficulty (for about 5 min in total): The first time with two words and the corresponding movements, the second time with an additional word and an additional movement and the third time with the same three words as the second time but with different movements for the first two words. The repetition with new words and changed corresponding movements was crucial in this exercise because children had to update the new information, shift between the different words and the corresponding movements, and inhibit the no longer correct movements from the previous round.

The second game was a version of playing tag. Children had to lie on the floor of the gymnasium in pairs, facing each other. The investigator had four balls with different colors in a box. At the beginning, two different colors were used. The investigator showed one of these two colors which acted as a cue. Depending on the color, either the child at the left or right side had to catch the other child before this child reached the wall of the gymnasium. After about 3 min, another color was introduced. When this new color was shown, children had to stay in place and roll around sideways. Again after about 3 min, a fourth color was introduced to prompt children to touch all four walls of the gymnasium in pairs as fast as possible. At the same time as the fourth color was introduced, the movement for the third color was changed (spinning around sitting instead of rolling around lying on the floor). This exercise required mainly shifting abilities, since children had to react appropriately to different rules. In addition, it required inhibition (children had to stay on the floor instead of jumping up when the third color was shown) and updating (new rule for the third color in the last round).

For the third game, different objects (rope, club, ball, rod, hula-hoop) were spread out over the gymnasium floor. One child from each pair had to balance on a moving object while observing his/her peer, who was jumping over the objects in an order of his/her own choice. Suddenly, the investigator interrupted the jumping, and the balancing child had to jump over the last three objects her/his peer jumped over before being interrupted. In this game, the balancing child had to update the order of the objects continuously.

The children attending the control condition made themselves comfortable on a mat and listened to an age-appropriate story. The story stopped after 15 min and in the remaining 5 min children answered some easy comprehension questions about the story.

### Instruments

*EFs* were measured by two computer-based tasks using E-Prime Software (Psychology Software Tools, Pittsburgh, PA). Each task took about 10 min to complete. The order of the two tasks was counterbalanced between participants. *Updating* was assessed with a computerized pictorial updating task which was adapted from Lee et al. (2011, 2012). Children were first presented with a picture of the animals included in the task and asked to name them to check whether the child was familiar with all of them. Then, several pictures of animals were shown one after another for 1900 ms each with an inter-stimulus interval of 100 ms. Children were asked to remember the last (1-back), the last two (2-back), or the last three (3-back) animals and to name them in the presented order, as soon as a question mark appeared on the screen. Because the number of animals differed in each trial, children could not anticipate the total number of animals. Therefore, they were obliged to continuously update the animals shown last. The practice trials were constructed with an increasing level of difficulty to make sure that the children understood the task correctly. The children completed three 1-back, two 2-back, and two 3-back practice trials. Error rate (one or more errors per trial) was 10.41% over all practice trials with the highest error rate in the 3-back task (36.11%). An accuracy threshold was not employed, however, through the individual testing it was possible to make sure that all children understood the task correctly by repeating the instruction as many times as necessary. After the practice trials, the children completed eight 3-back test trials in which four to seven animals were presented. For each animal they remembered, they received a point. If they remembered all three animals and named them in the correct order, they were credited with an additional point. Therefore, a maximum of 32 points could be gained in this task. The total number of points was used as the dependent measure.

To minimize confounding effects of elapsed time after the intervention, the cognitive testing had to be as brief as possible. Therefore, the measurement of *inhibition* and *shifting* were both included in one single modified Flanker task (Eriksen and Eriksen, 1974; Diamond et al., 2007; Röthlisberger et al., 2011). In this task, five fish were presented in a row on a screen and the child had to feed the target fish by pressing an external response button on the side where the fish had its mouth. The task consisted of four blocks. In the “pure” block, the fish were red and the target fish was situated in the middle. The block consisted of 10 congruent trials (target and flanking fish swimming in the same direction). The “standard” block consisted of 24 trials, half of which were congruent and half were incongruent (flanking fish swimming in the other direction than the target fish). In the third, “reversed” block, the fish were presented in a different color (yellow) and the children were instructed to feed the *flanking* fish and no longer the fish in the middle. This block consisted of 10 trials and was only used as practice for the following task. Data from this block was not included in the analyses. The fourth “mixed” block combined both previous conditions (red and yellow fish), i.e., the children had to switch between the two rules whenever the color of the fish changed. The mixed block consisted of 24 trials, 12 red and 12 yellow. Congruent and incongruent trials were presented randomly. To check whether the participants understood the task, they completed five practice trials before each block and were lead into a feedback loop with additional practice trials if performance was below 60% accuracy. Such an additional practice round was completed in only 9.8% of all blocks (collapsed across all participants and all measurement points); with the highest frequencies of feedback loops in the mixed block (14.7%). Inter-stimuli-intervals varied randomly from 800 to 1400 ms. The conflict score between trials with the highest rate of distraction (incongruent trials standard block) and trials with the lowest rate of distraction (congruent trials pure block) was calculated as the dependent measure for inhibition (Rueda et al., 2005; Fatzer and Roebers, 2012). For shifting, global switch costs were calculated (Chevalier and Blaye, 2009). Since trials in the mixed block not only required the child to shift between different tasks, but also contained inhibitory demands, the difference between the mixed and the standard block was calculated to control for the inhibition component.

### Background variables

Between post-test and follow-up, information about the following background variables was gathered: SES, activity level, body mass index (BMI), and enjoyment. The *SES* was assessed with the Family Affluence Scale II (FAS II; Boudreau and Poulin, 2009). The internal consistency for the four questions was relatively low (Cronbach's alpha = 0.48), but could not be enhanced by excluding one or more of the questions. The FAS II consists of four questions inquiring information about “the number of cars owned by the family; whether the participant has his/her own bedroom; number of yearly holidays taken by the family; and number of computers owned by the family” (Boudreau and Poulin, 2009). The answers to these questions were aggregated to a prosperity index ranging from 0 to 9. *Activity level* was assessed with a question addressing the number of days children practice any sportive activities in addition to physical education at school. The *BMI* was calculated as the body weight (in kg) divided by the square of the height (in m). *Enjoyment* was assessed with three questions: (1) *“How much did you like the activity at the gymnasium today?”* (2) *“How comfortable did you feel in the group you were with at the gymnasium?”* (3) *“How much did you like to do the computer tasks?”* The questions had to be answered on a 5-point Likert-scale with smileys (1 = sad smiley/frowny, 3 = indifferent smiley, 5 = happy smiley). The internal consistency for the three questions was relatively low (Cronbach's alpha = 0.57), but could not be enhanced by excluding a question. The points of the three answers were aggregated.

*Academic achievement (math, language)* was rated by the teacher on a 5-point Likert-scale (1 = very below-average, 2 = below-average, 3 = average, 4 = above-average, 5 = very above-average). The rating referred to the general achievement in math and language in the current school year. As the correlation between math and language achievement was high (*r* = 0.67, *p* < 0.001), the two values were aggregated as an estimation of academic achievement.

*Heart rate* was measured during the intervention/control condition using Suunto Dual Comfort Belts®. These belts transmitted the children's heart rate wireless to a laptop, where the data was monitored and saved in real-time for each participant. The mean heart rate during the intervention or control condition, respectively, was used in the analyses.

*Cortisol* was obtained by saliva samples. Children were asked to chew on a cotton roll for about 1 min. Afterwards the cotton rolls were placed in a plastic tube (Salivette®) and stored at −20°C until analysis. The samples were sent to Dresden LabService GmbH (Germany) for analysis of cortisol concentration. Collection of saliva samples is non-invasive and stress free and therefore particularly suitable for steroid measurements in children. Correlations between salivary and serum cortisol are high, confirming the validity of salivary measures of cortisol for determining the active free fraction of serum cortisol concentration (Thomas et al., 2009; Gatti and De Palo, 2011).

*Motor fitness* was assessed during a physical education lesson 2–4 weeks after the intervention using three different tests. The *20 meter shuttle run test* was used to estimate aerobic endurance performance (Léger et al., 1988), a *20-meter sprint* was used to estimate speed of action (Bös et al., 2009) and *jumping side to side* in two 50 × 50 cm squares as fast as possible was used to estimate coordination while pressed for time (Bös et al., 2009). The Cronbach's alpha of the three tests was 71. Evidence for reliability and validity for the shuttle run test has been provided by Liu et al. (1992) and McVeigh et al. (1995), for the 20-meter sprint by Bös (2001), and for the jumping side to side by Bös et al. (2004). The *z*-standardized values of the three tests were aggregated.

### Statistical analyses

For the outlier-analysis values which deviated more than three standard deviations from the mean (1.07%) were replaced by *M* ±3 *SD*. Z-standardization was conducted within the respective groups (EG and CG). Independent *t*-tests or Mann-Whitney tests were used to compare groups according to background variables, heart rate, enjoyment, cortisol concentration, and EF-performance at pre-test. Cohen's *d* was calculated as effect size.

Because there was an expected ceiling effect concerning accuracy in the Flanker task (mean accuracy was between 79 and 98%; see Appendix A for raw data), the mean reaction times were included in subsequent analyses. Performances in the different dependent variables did not or only weakly correlate with each other (*r* between 0.02 and 0.27). Therefore, the measures were not aggregated to one superior EF dimension.

For the main analysis, Group (CG, EG) × Time (pre-test, post-test, follow-up) repeated measures analyses of variance (ANOVA) were used to determine main and interaction effects of group and time for each of the EF subdimensions and for the cortisol level. Level of significance was set at *p* < 0.05 for all analyses. Partial eta square (η^2^_*p*_) was reported as an estimation of effect size. When sphericity was violated in the ANOVA, the Greenhouse-Geisser correction was applied.

## Results

### Preliminary analyses

As a manipulation check, the EG and the CG were compared with regard to the heart rate during the intervention or the control condition. As expected, the heart rate was significantly higher in the EG (*M* = 156.76, *SD* = 14.09) than in the CG [*M* = 89.66, *SD* = 9.33; *t*_(102)_ = 28.74, *p* < 0.001, *d* = 5.62], thus establishing the increased physical effort in the intervention compared to the control condition. The mean heart rate in the experimental group corresponds to moderate to vigorous physical activity (Ainsworth et al., 1993). The enjoyment did not differ between groups (*U* = 1328.00, *z* = −0.18, *p* = 0.86, *d* = 0.06). Furthermore, neither differences in cognitive performance [updating: *t*_(102)_ = −0.44, *p* = 0.66, *d* = 0.09; inhibition: *t*_(102)_ = 1.03, *p* = 0.31, *d* = 0.20; shifting: *t*_(100)_ = 1.06, *p* = 0.29, *d* = 0.21] nor in cortisol level [*t*_(99)_ = −0.80, *p* = 0.43, *d* = 0.16] were found between the groups in the pre-test session. As expected, there was a significant main effect for congruency [*F*_(1, 103)_ = 40.04, *p* < 0.001, η^2^_*p*_ = 0.28], with longer reaction times for incongruent than congruent trials. The main effect for switching [*F*_(1, 101)_ = 1.11, *p* = 0.29, η^2^_*p*_ = 0.01] was not significant and therefore all trials in the mixed block were entered in the calculation of switch-costs. The order of the two tasks did not influence the results, therefore task order was not further considered in the analyses. There were no significant group differences evident in terms of the background variables (sex, age, SES, BMI, activity level, estimated academic achievement, and motor fitness). Therefore, none of these variables were included as a covariate in subsequent analyses. As an aside, there were no gender differences for EF performance and cortisol concentration at all three measurement points. To check if there was a necessity to control for specific background variables in the following analyses including cortisol, correlations of cortisol with age, SES and heart rate were calculated (variables which may theoretically influence both, cortisol level and cognitive functioning). No significant correlations were found neither in the EG (*r* between 0.02 and 0.23) nor in the CG (*r* between 0.05 and 0.18). Thus, we did not control for any background variable in the following analyses.

### Cognitive effects of the intervention

**Figure 2:**
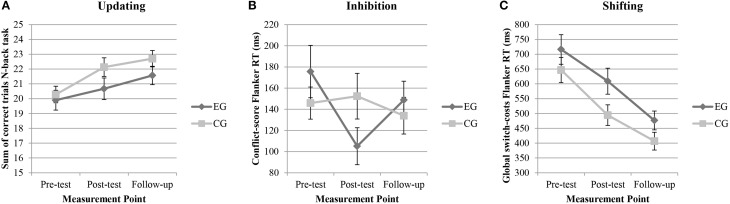
**Representation of means and error bars (representing standard error of the mean) for (A) updating, (B) inhibition, and (C) shifting over the three measurement points (pre-test, post-test, follow-up)**. *CG*, control group, *EG*, experimental group, *RT*, reaction time.

Figure 2 presents mean performance for the three dependent variables (updating, inhibition, and shifting) at pre-test, post-test, and follow-up. Of specific interest were interaction effects. The Group × Time interaction was significant for *inhibition* only [*F*_(2, 204)_ = 3.89, *p* = 0.02, η^2^_*p*_ = 0.04]. Tests of within-subjects contrasts revealed that the EG improved more from pre- to post-test than the CG [*F*_(1, 102)_ = 6.04, *p* = 0.02, η^2^_*p*_ = 0.06; Figure 2B]. The interaction effect remained significant between post-test and follow-up [*F*_(1, 102)_ = 5.06, *p* = 0.03, η^2^_*p*_ = 0.05], indicating, however, a decline of the benefits in the EG compared to the CG. No significant Group × Time interaction effects were found for updating [*F*_(2, 204)_ = 0.80, *p* = 0.45, η^2^_*p*_ = 0.01] and shifting [*F*_(1.7, 170.43)_ = 0.43, *p* = 0.65, η^2^_*p*_ = 0.004] (Figures 2A,C).

To address the question of individual differences in response to the intervention, correlations between the improvement from pre- to post-test in inhibition (difference scores) in the EG and the background variables were calculated. None of the correlations reached significance (age: *r* = −0.23, *p* = 0.16; BMI: *r* = 0.01, *p* = 0.92; activity level: *r* = −0.02, *p* = 0.90; SES: *r* = 0.13, *p* = 0.38, motor fitness: *r* = −0.19, *p* = 0.21; academic achievement: *r* = −0.07, *p* = 0.64), suggesting that the positive impact of physical activity on inhibition was independent of any of these specific characteristics of participants.

The main effect of time was significant for updating [*F*_(2, 204)_ = 11.50, *p* < 0.001, η^2^_*p*_ = 0.10] and shifting [*F*_(1.7, 170.43)_ = 47.62, *p* < 0.001, η^2^_*p*_ = 0.32], referring to an improvement in both groups (Figures 2A,C). Tests of within-subject contrasts revealed that from pre- to post-test the main effect of time was significant in both, updating [*F*_(1, 102)_ = 10.24, *p* = 0.002, η^2^_*p*_ = 0.09] and shifting [*F*_(1, 100)_ = 32.13, *p* < 0.001, η^2^_*p*_ = 0.24]. From post-test to follow-up, however, the main effect of time remained significant only in shifting [*F*_(1, 100)_ = 25.01, *p* < 0.001, η^2^_*p*_ = 0.20; updating: *F*_(1, 102)_ = 3.22, *p* = 0.08, η^2^_*p*_ = 0.03]. Thus, there is evidence to suggest a practice effect in these tasks, especially between the first and the second measurement point. The main effect of group did not reach significance in any EF subdimension [updating: *F*_(1, 102)_ = 1.78, *p* = 0.17, η^2^_*p*_ = 0.01; inhibition: *F*_(1, 102)_ = 0.00, *p* = 0.97, η^2^_*p*_ < 0.001; shifting: *F*_(1, 98)_ = 2.26, *p* = 0.14, η^2^_*p*_ = 0.02].

### Physiological effects of the intervention

**Figure 3:**
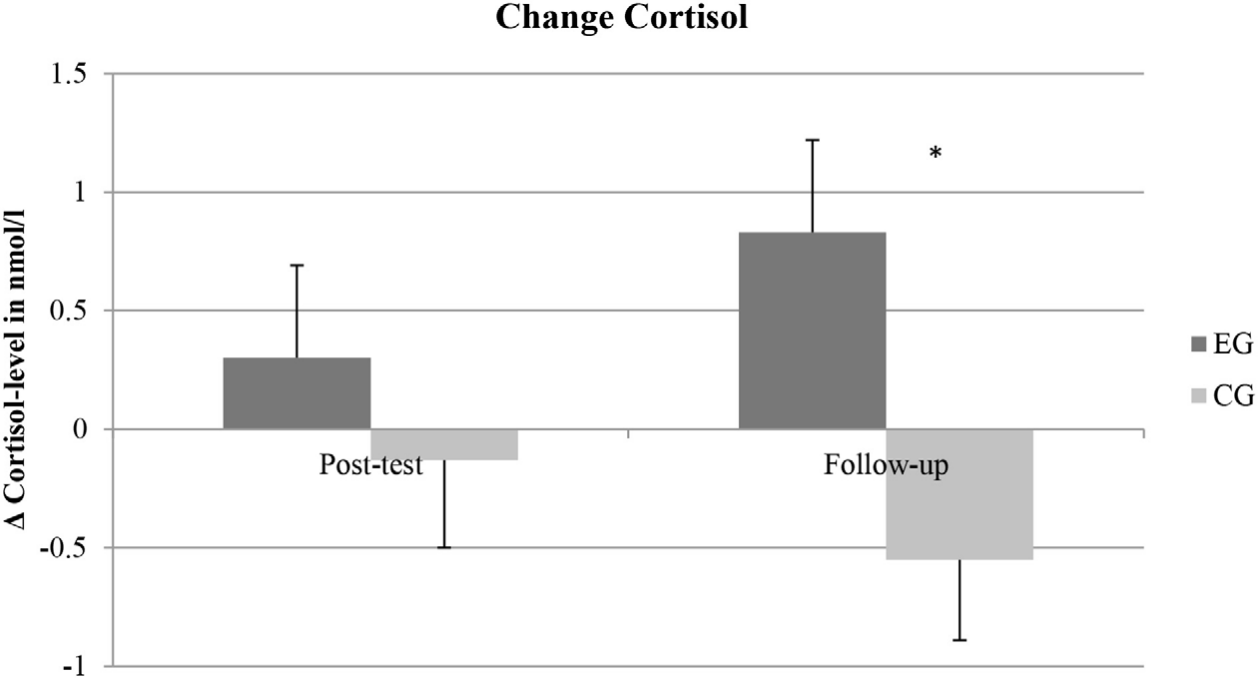
**Representation of means and error bars (representing standard error of the mean) for the Δ in Cortisol level in nmol/l compared to pre-test**. *EG*, experimental group, *CG*, control group. ^*^p < 0.05.

To examine changes in cortisol level an ANOVA with repeated measures was conducted. The interaction between time and group was significant [*F*_(2, 196)_ = 3.69, *p* = 0.03, η^2^_*p*_ = 0.04], in absence of main effects of time [*F*_(2, 196)_ = 0.15, *p* = 0.86, η^2^_*p*_ = 0.002] or group [*F*_(1, 98)_ = 0.64, *p* = 0.43, η^2^_*p*_ = 0.006]. Tests of within-subjects contrasts revealed that the cortisol level in the EG increased more strongly between post-test and follow-up than in the CG [*F*_(1, 98)_ = 4.14, *p* < 0.05, η^2^_*p*_ = 0.04]. No significant interaction between time and group was found between pre- and post-test [*F*_(1, 98)_ = 0.49, *p* = 0.49, η^2^_*p*_ = 0.005]. As can be seen in Figure 3, cortisol concentration seems to increase in the EG and decrease in the CG. However, only the overall increase in the EG from pre-test to follow-up (18.15%) reached significance [*t*_(49)_ = −2.07, *p* = 0.04, *d* = 0.35] and differed significantly from the CG [*t*_(98)_ = 2.60, *p* = 0.01, *d* = 0.52].

As a next step, analyses exploring the relationship between (changes in) cortisol concentration and inhibition performance were conducted. Cortisol level correlated significantly with inhibition performance only at post-test (*r* = −0.21, *p* = 0.03) and not at pre-test (*r* = 0.02, *p* = 0.86) or follow-up (*r* = 0.07, *p* = 0.50). When the two groups (EG and CG) were considered separately, the correlation between cortisol level and inhibition performance at post-test was only significant in the EG (*r* = −0.29, *p* = 0.04) and not in the CG (*r* = −0.16, *p* = 0.25). Furthermore, the change in cortisol level between pre- and post-test correlated significantly with the inhibition performance at post-test in the EG (*r* = −0.33, *p* = 0.02) but not in the CG (*r* = −0.13, *p* = 0.35). Change in cortisol concentration between post-test and follow-up did not correlate with inhibition performance at follow-up neither in the EG (*r* = 0.10, *p* = 0.50) nor in the CG (*r* = 0.17, *p* = 0.13). The pattern of results did not change when controlling for heart rate as a measurement of the intervention intensity or when controlling for inhibition performance at pre-test or at post-test, respectively.

**Figure 4:**
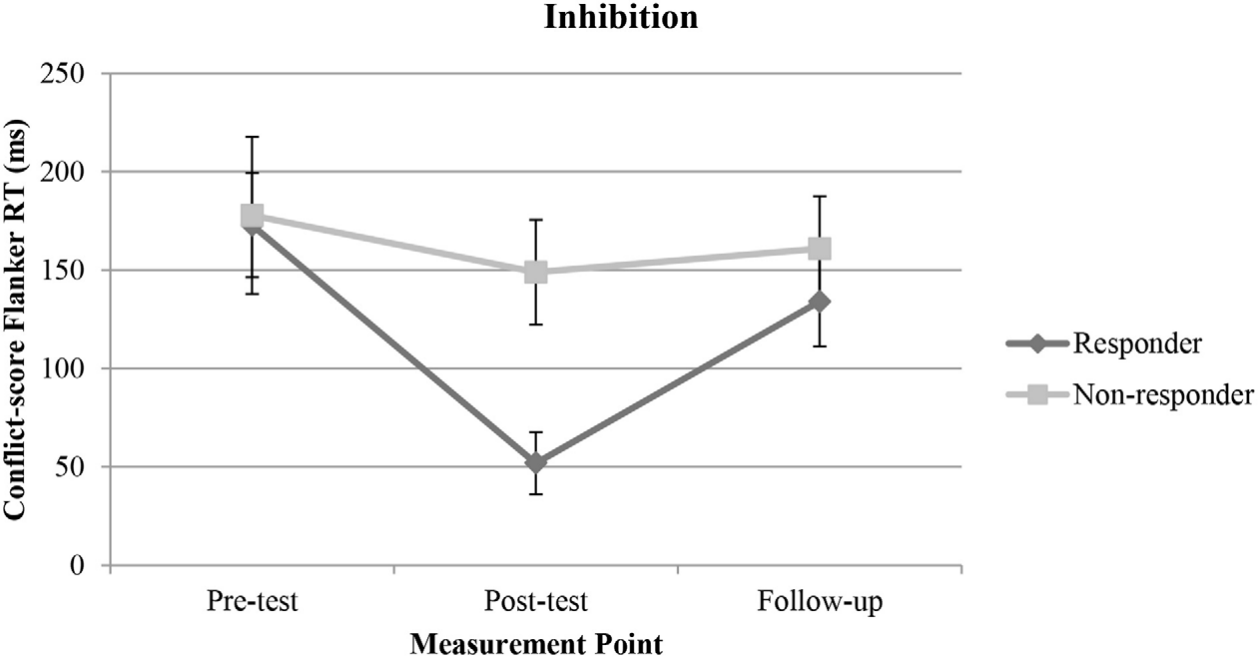
**Representation of means and error bars (representing standard error of the mean) for inhibition in the responder (subjects showing a cortisol elevation between pre- and post-test) and in the non-responder (subjects showing no or a negative change in cortisol level between pre- and post-test) group within the experimental group over the three measurement points (pre-test, post-test, follow-up)**. *RT*, reaction time.

Inspection of the distribution of cortisol change in the EG motivated us to explore possible differential effects on inhibition within the EG as a function of cortisol increase. We therefore divided the EG into two groups. Participants with a positive change in cortisol level between pre- and post-test were assigned to one group (the “responder group,” *n* = 23) and participants with no change or a decrease in cortisol level between pre- and post-test were assigned to the other group (the “non-responder group,” *n* = 28). The change in cortisol differed significantly between the responder (*M* = 2.66, *SD* = 2.20) and the non-responder group [*M* = −1.60, *SD* = 1.29; *t*_(48)_ = −8.55, *p* < 0.001, *d* = −2.36]. The two groups did not differ in their mean heart rate [responder: *M* = 156.65, *SD* = 13.08; non-responder: *M* = 156.86, *SD* = 15.11; *t*_(49)_ = 0.05, *p* = 0.96, *d* = 0.01]. An ANOVA with repeated measures was conducted within the EG with a 2 (responder, non-responder) × 3 (pre-test, post-test, follow-up) design. The interaction between time and group was significant [*F*_(2, 98)_ = 2.52, *p* = 0.04, η^2^_*p*_ = 0.05]. The main effect of time was also significant [*F*_(2, 98)_ = 6.25, *p* = 0.003, η^2^_*p*_ = 0.11], in absence of a main effect of group [*F*_(1, 49)_ = 1.81, *p* = 0.18, η^2^_*p*_ = 0.03]. Tests of within subjects contrasts revealed a significant interaction between pre- and post-test [*F*_(1, 49)_ = 3.90, *p* = 0.03, η^2^_*p*_ = 0.07] and between post-test and follow-up [*F*_(1, 49)_ = 3.67, *p* = 0.03, η^2^_*p*_ = 0.07], indicating a significant stronger improvement in inhibition performance between pre- and post-test in the responder group compared to the non-responder group (Figure 4). The benefits in the responder group at post-test, however, dropped back to the baseline at follow-up.

## Discussion

The aim of the present study was to investigate immediate and delayed effects of acute physical activity that included cognitive engagement on children's different EF subdimensions and on their cortisol concentration. Overall, the findings revealed that this special form of acute physical activity affected inhibition positively, but did not substantially impact updating and shifting. Furthermore, the intervention seems to result in an increased cortisol level and this change in cortisol level seems to partly predict inhibition performance.

To the best of our knowledge, this study was the first to include measures of all three subdimensions of EFs. This allowed us to explore the possibility that one EF subdimension would be more prone to be positively affected by the same acute physical activity than others. In fact, only inhibition was affected through acute physical activity whereas updating and shifting were not. This result is in line with a review that includes mainly studies with adult participants and indicates that updating and shifting are less easily affected by acute physical activity, compared to inhibition (Barenberg et al., 2011). It is also consistent with results in children and adolescents that indicate positive effects on inhibition (Hillman et al., 2009; Kubesch et al., 2009; Best, 2012; Drollette et al., 2012; Pontifex et al., 2012), but no effects on updating and shifting (Tomporowski et al., 2008a; Kubesch et al., 2009; Budde et al., 2010a; Drollette et al., 2012). Thus, acute physical activity seems to enhance inhibition in elementary school children, which in turn may facilitate children's selective and focused attention, a skill which seems to be crucial for school achievement (Diamond, 2012).

In contrast to most previous studies, our acute physical activity intervention included cognitive engagement. Results showed that this form of acute physical activity seems to have similar effects as endurance-based interventions but additionally bears the advantage of being somewhat more child-appropriate due to the game character. However, as we did not compare different forms of acute physical activity interventions, caution is warranted with respect to firm interpretations concerning different forms of physical interventions. In sum, our study confirms and extends the literature by showing that acute physical activity including cognitive engagement may have beneficial effects on inhibition in children, whereas effects on updating and shifting seem to be more difficult to obtain.

The physiological processes at work are still unclear. We examined cortisol as a possible underlying mechanism in the current approach. In fact, there was a significantly greater increase in cortisol concentration in the intervention group compared to the resting control group. In line with previous findings, showing that the peak of cortisol concentration was recorded 20–30 min after acute physical activity (Kirschbaum and Hellhammer, 1994; Daly et al., 2005), the elevation was most pronounced between post-test and follow-up. In addition, there was a significant correlation between change in cortisol between pre- and post-test and inhibition performance at post-test, controlling for inhibition performance at pre-test. Thus, the change in cortisol between pre- and post-test seems to predict inhibition performance in the EG at post-test, at least to some extent. Furthermore, children participating in the intervention classified as responders, i.e., their cortisol concentration increased between pre- and post-test, improved their inhibition performance significantly more between pre- and post-test than non-responders. These results possibly suggest that an increase in cortisol level between pre- and post-test is related to better inhibition performance at post-test. The additional increase in cortisol level between post-test and follow-up, however, did not enhance children's performance any further. Thus, results seem to support the assumed inverted U-shaped relationship between cortisol and inhibition performance with better performance after a moderate elevation in cortisol, compared to lower and higher levels of cortisol (Erickson et al., 2003; Blair et al., 2005). However, caution is warranted with this interpretation since the cortisol elevation in the EG between pre- and post-test did not reach significance. In addition, the significant time effect in the ANOVA comparing the responder and the non-responder group indicates a similar pattern of results, i.e., improvement in inhibition between pre- and post-test followed by deterioration in inhibition between post-test and follow-up, in both groups. Therefore, other processes in addition to cortisol release seem to be involved in improving inhibition performance and cortisol release might enhance the positive effect, but is maybe not crucial for an improvement in inhibition.

Cortisol release could also just come along with other physiological processes such as the release of nerve growth factors, brain-derived neurotrophic factors, and/or neuroendocrinological substances (Hollmann and Strüder, 2000; Cahill and Alkire, 2003; Gold et al., 2003; Vaynman et al., 2004; Ferris et al., 2007; Winter et al., 2007), without affecting these processes and cognitive performance directly. In this manner, cortisol would be a form of marker for ongoing physiological processes caused by an activation of the sympathetic nervous system. Because of the delay in cortisol release (Kirschbaum and Hellhammer, 1994; Daly et al., 2005), an increased concentration at follow-up signifies an activation of the sympathetic nervous system 20–60 min earlier, a time frame that included the post-test session. Thus, instead of influencing cognitive performance directly, an elevation of cortisol concentration may indicate an antecedent activation of the sympathetic nervous system and therewith the release of other neuroendocrinological substances with shorter delays of release.

Another explanation for the relationship between change in cortisol concentration and inhibition performance at post-test would be that the increase in cortisol level between pre- and post-test reflects the intensity by which children completed the intervention or control condition, respectively. However, since there was no significant correlation between heart rate and cortisol within the EG and the CG and since the responder and the non-responder group did not differ in their heart rate, this explanation seems unlikely. In sum, there appears to be some kind of relationship between change in cortisol concentration and inhibition performance but the exact role of cortisol in the relationship between acute physical activity and cognitive performance is still unclear.

We had a hard time interpreting the selective effect of acute physical activity on inhibition. For one, the different subdimensions of EF can be differentially affected depending on an individual's development. Inhibition, in this context, may be seen as the EF subdimension that develops earlier than the others and may therefore be more easily targeted in young elementary school children. Second, the sensitivity of the inhibition measurement may have been superior compared to the other two indicators, making the detection of inhibition effects more likely. But, as also updating and switching proved to document performance increases between the measurement points (that may be attributable to practice), measurement issues seem improbable. A third possible interpretation concerns the suggestion of specific neurotransmitter systems being related to specific EF subdomains (Montgomery et al., 2005; Verdejo-García and Pérez-García, 2006). However, since there is no clear evidence that short-term elevated cortisol concentration affects only one specific neurotransmitter system, such as serotonin, this interpretation is also unlikely, unless serotonin is released independently from cortisol. Definitely, more research is needed to address the specific nature of the cortisol-cognitive performance link including the question regarding specific or general mechanisms underlying the link between acute physical activity and cognitive performance.

Another major finding in the present study was that cognitive benefits in inhibition were found immediately (0–10 min) and 10–20 min (no task order effects), but no longer 40 min after acute physical activity. In the only study also assessing EFs twice after acute physical activity in children, the same pattern of results with improved performance in inhibition immediately after the activity but no longer after 45 min was reported (Kubesch et al., 2009). In another study (Hillman et al., 2009) the cognitive testing did not begin until 25 min after acute physical activity but improved inhibition performance was still observable. Together with results of studies with adults (Joyce et al., 2009; Pontifex et al., 2009), these results indicate that positive effects may persist over approximately 30 min but then drop back to baseline. The practical significance of this finding is that several insertions of short bouts of physical activity during a school day may be beneficial for supporting children's inhibition. Even though the positive effect might not last a whole lesson, no detrimental effects seem to occur and enhanced inhibition can be expected at least for the first half of the lesson after acute physical activity. Based on the present study, however, it is not possible to conclude how long the effects persist exactly. Furthermore, it is unclear whether the duration of the intervention influences the continuance of potential effects. To determine the continuance of effects, future studies should therefore vary the timing of post-tests and the duration of the intervention systematically.

Of course, the present approach also has some limitations. One limitation is the timing and number of the cognitive assessment and the cortisol measurement. Since there is evidence that the peak of cortisol concentration occurs 20–30 min after acute physical activity (Kirschbaum and Hellhammer, 1994; Daly et al., 2005), an additional measurement of cortisol and cognitive performance within this time frame would be interesting. However, the cortisol concentration was significantly higher in the EG compared to the CG 40 min after acute physical activity, confirming previous findings in adults showing that cortisol concentration seems to continue to be elevated 60 min after acute physical activity (Daly et al., 2005). Nevertheless, more measurement points of cortisol would be desirable in future studies to gain more detailed information about ongoing processes. Furthermore, an assessment of children's performance during the physical activity intervention would be informative because there may be an optimal challenge point depending on the joint moderating effect of the complexity of the movement task and the children's individual skill level (Pesce et al., 2013). However, the intervention was designed in collaboration with three teachers to make sure that the cognitive and physical requirements were in line with the skills of the assessed age group and observations through the investigator confirmed that the activities were challenging but manageable for the children. Another limitation is the assessment of the EF subdimensions with only one task each. However, since we wanted to investigate immediate effects of acute physical activity on EF performance, the cognitive testing needed to be as brief as possible in order to minimize confounding effects of elapsed time and fatigue after the intervention. Therefore, it was not possible to include more than one task per subdimension. Despite this disadvantage, the inclusion of three EF subdimensions increased our knowledge about the vulnerability of different executive processes, with inhibition appearing to be especially prone to positive effects through physical activities. Furthermore, the stability of potential effects should be examined more systematically, especially because this issue is important for theory and practice. A systematic variation of intervention content and duration could help to determine which factors are essential for an improvement of EF performance. Furthermore, it could point to mechanisms underlying the link between acute physical activity and cognitive performance. In addition, more and more detailed physiological measurements are required to understand the mechanisms underlying the relationship between acute physical activity and cognitive performance.

Taken together, our study extends existing findings which included mainly endurance-oriented interventions because we were able to show that a similar pattern of results can be obtained conducting an acute physical activity intervention including cognitive engagement. Activities similar to our intervention are more suitable for young children due to their game character and they are applicable in normal physical education lessons at school. From this perspective, our approach has strong practical relevance: the beneficial effect of this form of acute physical activity seemed to be independent of specific characteristics of participants such as age, motor fitness, academic achievement, and others. Thus, although the effect of acute physical activity on inhibition was only small, it seems to be practically relevant as it indicates that positive effects can be expected in a broad range of normatively developing children.

### Conflict of interest statement

The authors declare that the research was conducted in the absence of any commercial or financial relationships that could be construed as a potential conflict of interest.
