# Supplementary material for: Cognitive and physiological effects of an acute physical activity intervention in elementary school children
Source: Front Psychol. 2014 Dec 18;5:1473. doi: 10.3389/fpsyg.2014.01473 (PMC4270126; doi:10.3389/fpsyg.2014.01473)
Supplement: Supplementary file 1 [file DataSheet1.DOCX]

Appendix A

*Means and standard deviations for accuracy in the Flanker task at the three measurement points (pre-test, post-test, follow-up) for the experimental and the control group and F-ratios and degrees of freedom of one-way independent analyses of variance comparing the two groups.*

|  | |  | Accuracy  (No. of correct responses)  *M (SD)* | |  |
| --- | --- | --- | --- | --- | --- |
| Trials | |  | EG | CG | *F (df)* |
| Pure (congruent) | | |  |  |  |
|  | Pre-test | | 9.27 (.92) | 9.36 (.83) | .24 (1, 103) |
|  | Post-test | | 9.61 (.75) | 9.55 (.67) | .19 (1, 103) |
|  | Follow-up | | 9.73 (.53) | 9.70 (.61) | .06 (1, 103) |
|  |  | |  |  |  |
| Standard (congruent) | | |  |  |  |
|  | Pre-test | | 11.27 (1.66) | 11.60 (.71) | 1.74 (1, 103) |
|  | Post-test | | 11.67 (.55) | 11.60 (.69) | .26 (1, 103) |
|  | Follow-up | | 11.71 (1.04) | 11.75 (.55) | .09 (1, 103) |
|  |  | |  |  |  |
| Standard (incongruent) | | |  |  |  |
|  | Pre-test | | 11.18 (.97) | 11.26 (.88) | .23 (1, 103) |
|  | Post-test | | 11.43 (.83) | 11.51 (.70) | .27 (1, 103) |
|  | Follow-up | | 11.45 (1.15) | 11.68 (.64) | 1.57 (1, 103) |
|  |  | |  |  |  |
| Mixed (non-switch) | | |  |  |  |
|  | Pre-test | | 9.78 (1.06) | 9.50 (1.48) | 1.21 (1, 101) |
|  | Post-test | | 9.75 (1.29) | 9.83 (1.19) | .12 (1, 103) |
|  | Follow-up | | 9.92 (1.06) | 9.75 (1.14) | .60 (1, 103) |
|  |  | |  |  |  |
| Mixed (switch) | | |  |  |  |
|  | Pre-test | | 10.78 (1.23) | 10.62 (1.16) | .48 (1, 101) |
|  | Post-test | | 10.94 (1.01) | 10.75 (1.18) | .75 (1, 103) |
|  | Follow-up | | 10.96 (.94) | 10.96 (1.24) | .00 (1, 103) |

*Note*. The one-way independent analyses of variance revealed no significant group differences. *CG* = control group, *EG* = experimental group.

Appendix B

*Means and standard deviations for reaction time in the Flanker task at the three measurement points (pre-test, post-test, follow-up) for the experimental and the control group and F-ratios and degrees of freedom of one-way independent analyses of variance comparing the two groups.*

|  | |  | Reaction time  (in msec)  *M (SD)* | |  |
| --- | --- | --- | --- | --- | --- |
| Trials | |  | EG | CG | *F (df)* |
| Pure (congruent) | | |  |  |  |
|  | Pre-test | | 534 (140) | 536 (123) | .01 (1, 103) |
|  | Post-test | | 546 (125) | 552 (131) | .06 (1, 103) |
|  | Follow-up | | 553 (102) | 574 (155) | .61 (1, 103) |
|  |  | |  |  |  |
| Standard (congruent) | | |  |  |  |
|  | Pre-test | | 681 (203) | 649 (162) | .73 (1, 103) |
|  | Post-test | | 624 (139) | 661 (211) | .97 (1, 103) |
|  | Follow-up | | 679 (163) | 663 (185) | .23 (1, 103) |
|  |  | |  |  |  |
| Standard (incongruent) | | |  |  |  |
|  | Pre-test | | 710 (233) | 682 (169) | .49 (1, 103) |
|  | Post-test | | 651 (147) | 704 (217) | 2.13 (1, 103) |
|  | Follow-up | | 703 (175) | 708 (196) | .02 (1, 103) |
|  |  | |  |  |  |
| Mixed (non-switch) | | |  |  |  |
|  | Pre-test | | 1352 (430) | 1288 (413) | .30 (1, 101) |
|  | Post-test | | 1240 (384) | 1159 (327) | 1.34 (1, 103) |
|  | Follow-up | | 1154 (307) | 1074 (280) | 2.08 (1, 103) |
|  |  | |  |  |  |
| Mixed (switch) | | |  |  |  |
|  | Pre-test | | 1470 (561) | 1328 (470) | 2.34 (1, 101) |
|  | Post-test | | 1254 (410) | 1185 (323) | .89 (1, 103) |
|  | Follow-up | | 1186 (339) | 1126 (360) | .92 (1, 103) |

*Note*. The one-way independent analyses of variance revealed no significant group differences. *CG* = control group, *EG* = experimental group.

Appendix C

*Means and standard deviations for Cortisol concentration in nmol/l at the three measurement points for the experimental and the control group and F-ratios and degrees of freedom of one-way independent analyses of variance comparing the two groups.*

|  | Cortisol concentration in nmol/l  *M (SD)* | |  |
| --- | --- | --- | --- |
|  | EG | CG | *F (df)* |
| Pre-test | 4.57 (1.81) | 4.88 (2.09) | .64 (1, 99) |
| Post-Test | 4.87 (2.56) | 4.75 (2.64) | .06 (1, 100) |
| Follow-up | 5.40 (2.69) | 4.33 (1.73) | 5.76* (1, 100) |

*Note*. *CG* = control group, *EG* = experimental group.

**p* < .05.
